# Special Issue “Molecular Mechanisms of Cancer Invasion and Metastasis”

**DOI:** 10.3390/ijms27219919

**Published:** 2026-11-05

**Authors:** Zbigniew Baster

**Affiliations:** Laboratory for Cell and Tissue Engineering, Department of Biomedical Engineering, Eindhoven University of Technology, 5600 MB Eindhoven, The Netherlands; z.j.baster@tue.nl

In the past decades, substantial progress has been made in the detection and treatment of localized tumors. Nonetheless, metastatic disease remains responsible for most cancer-related deaths [1]. The formation of a metastasis is a multistep process in which cancer cells invade the surrounding tissue, enter and survive in the circulation, leave the blood or lymphatic vessels, and adapt to a distant organ [2,3,4,5]. Importantly, none of these stages are regulated by cancer cells alone. Cell adhesion, migration, metabolism, immune pressure, the extracellular matrix, and the properties of the target organ together determine whether disseminated cells will be eliminated, remain dormant, or form a secondary tumor.

The Special Issue “Molecular Mechanisms of Cancer Invasion and Metastasis” includes seven contributions, four original articles and three reviews, that examine this problem from different but complementary perspectives (Figure 1). The collection covers in vivo models, cell adhesion and mechanotransduction, organ-specific metastasis, mitochondrial quality control, inflammation-induced cell-state changes, purinergic metabolism in cancer stem-like cells, and links between epidemiological observations and molecular networks. Although the articles use different experimental systems and analytical methods, they share one important message: metastatic potential does not arise from one universal pathway, but from dynamic interactions between cellular programs and the environment in which they operate.

Martínez-López and López-Gil [6] describe the growing role of zebrafish (*Danio rerio*) in the study of cancer metastasis. The optical transparency of the model, its rapid development, and the possibility of combining genetic manipulation with xenografting make it possible to follow tumor cells and their interactions with the host in vivo. The review summarizes applications ranging from the analysis of invasion and vascular remodeling to immune evasion, metastatic dissemination, drug screening, and patient-derived xenografts. In this context, zebrafish provide a useful bridge between simplified cell culture experiments and mammalian models, especially when the dynamics of early metastatic events are important.

Changes in cell adhesion and migration contribute to cancer invasion and metastasis. In our review [7], we examined how talins and integrins regulate these processes through interactions between cancer cells and the extracellular matrix. We described their involvement in epithelial–mesenchymal transition, invadopodia formation, and matrix degradation, as well as mechanisms supporting cancer cell survival after detachment. Furthermore, we discussed how interactions with the tumor microenvironment influence cancer progression, how talin and integrin expression relates to patient prognosis, and how integrin signaling contributes to immune evasion and treatment resistance. These findings provide a basis for considering adhesion- and migration-related proteins as therapeutic targets. We summarized approaches targeting talins, integrins, and their associated pathways, with particular attention to migrastatic treatments aimed at limiting invasion and metastatic spread. Such treatments could complement therapies directed at tumor growth, although their development requires consideration of the functions these proteins also perform in healthy tissues.

The following stages of metastasis strongly depend on the tissue that cancer cells encounter. Jacome and colleagues [8] summarize the molecular underpinnings of brain metastases arising from lung and breast cancers, melanoma, and other primary tumors. The authors follow the metastatic process from tumor cell priming and survival in the circulation to crossing the blood–brain barrier and adaptation to the neural microenvironment. Some requirements are shared between cancer types, whereas others remain specific to the primary tumor. This heterogeneity helps to explain why brain metastases are difficult to treat and why therapeutic strategies must consider both the origin of the cancer cells and the biology of the target organ.

Mitochondrial homeostasis is another factor associated with cancer progression. Bian and colleagues [9] used LASSO and multivariable Cox analyses to identify a prognostic signature composed of four mitophagy-related E3 ubiquitin ligases: ARIH1, SIAH2, UBR5, and WWP2. The high-risk group showed poorer overall survival, while gene-expression analyses suggested altered mitochondrial metabolism, immune dysregulation, increased immune-checkpoint expression, and mitochondrial DNA stress associated with a senescence-associated secretory phenotype. Interestingly, the analysis also predicted higher sensitivity of high-risk tumors to compounds targeting the Akt/PI3K/mTORC1 axis. These results connect mitochondrial regulation with the immune and metabolic state of breast tumors, although prospective clinical validation and further functional studies will be required.

Cancer cell plasticity is also regulated by inflammatory signaling. García-Morales and colleagues [10] showed that interleukin-1β drives MCF-7 breast cancer cells towards a more aggressive mesenchymal-like phenotype, while cannabidiol promotes the recovery of epithelial characteristics and reduces invasive behavior. Quantitative proteomics identified 24 proteins regulated in opposite directions by interleukin-1β and cannabidiol. Network analysis further highlighted a BRCA1-MSN-CORO1A axis associated with the mesenchymal state and a SUPT16H-SETD2-H2BC12 axis associated with cannabidiol treatment. Together, these observations suggest that the reversal of an invasive phenotype may involve cytoskeletal as well as chromatin-related mechanisms. These observations also provide specific candidates for validation in more complex models.

The work of Valdivia and colleagues [11] focuses on extracellular adenosine metabolism in gastric cancer stem-like cells. Compared with differentiated MKN-74 cells, the stem-like population released more extracellular ATP and showed greater CD39-dependent ATP hydrolysis. Increased AMP hydrolysis was attributed mainly to CD73, and the cells also showed higher expression of the equilibrative nucleoside transporter ENT2. These changes favor the production and transport of extracellular adenosine, which can affect tumor cell survival and anti-tumor immune responses. Thus, the study identifies a possible metabolic vulnerability of gastric cancer stem-like cells. At the same time, any therapy targeting this pathway will need to preserve the physiological functions of purinergic signaling.

Finally, Górawski, Wicik, and Blecharz-Klin [12] combined epidemiological evidence with bioinformatic analyses to study the relationship between acetaminophen use and sex hormone-related cancers. The currently available population-based evidence does not consistently indicate an increased risk and, in some settings, suggests a possible protective association. Nevertheless, the molecular analysis identified acetaminophen targets and pathways related to ovarian and prostate cancers, together with possible links to breast cancer through drug metabolism genes. This work is a useful example of how population-level observations and molecular hypotheses can be analyzed within one framework. It also underlines that associations obtained from heterogeneous datasets should not be interpreted as evidence of causality without experimental and prospective validation.

Several connections between the contributions are particularly relevant to further research. Adhesion and matrix remodeling participate in both local invasion and the establishment of metastatic colonies, as illustrated by the talin–integrin and brain metastasis reviews [7,8]. Inflammation and metabolic changes provide additional mechanisms through which cells may alter their behavior and their environment [9,10,11]. Moreover, many of the pathways used by metastatic cells are not cancer-specific; they are modified versions of physiological mechanisms controlling migration, tissue remodeling, repair, and immune responses. It is therefore important to distinguish mechanisms that are required for normal function from those that create a selective vulnerability in metastatic cells.

The studies presented here also show the value of combining complementary methods. Live imaging and animal models reveal when and where metastatic events occur, while proteomics, gene expression analyses, and computational models identify candidate networks. Clinical and epidemiological data are then needed to determine whether these mechanisms remain relevant in patients and populations. In future studies, single-cell and spatial approaches, organoids, microphysiological systems, and organ-relevant in vivo models may help connect transient molecular states with stable metastatic phenotypes. Candidate signatures should also be validated in independent and prospectively collected cohorts before being considered for clinical application.

In this Editorial, I wanted to underline that metastasis is an adaptive process rather than the outcome of a single molecular switch. The articles included in this Special Issue move from zebrafish imaging and adhesion mechanics to brain organotropism, mitochondrial regulation, inflammatory plasticity, cancer stem-like-cell metabolism, and population-to-pathway analysis. Together, they expand the range of questions that can be addressed experimentally and identify several mechanisms that may become relevant for diagnosis or treatment. I believe that further integration of these perspectives will support the development of safer and more effective strategies for preventing and treating metastatic disease.

## Figures and Tables

**Figure 1:**
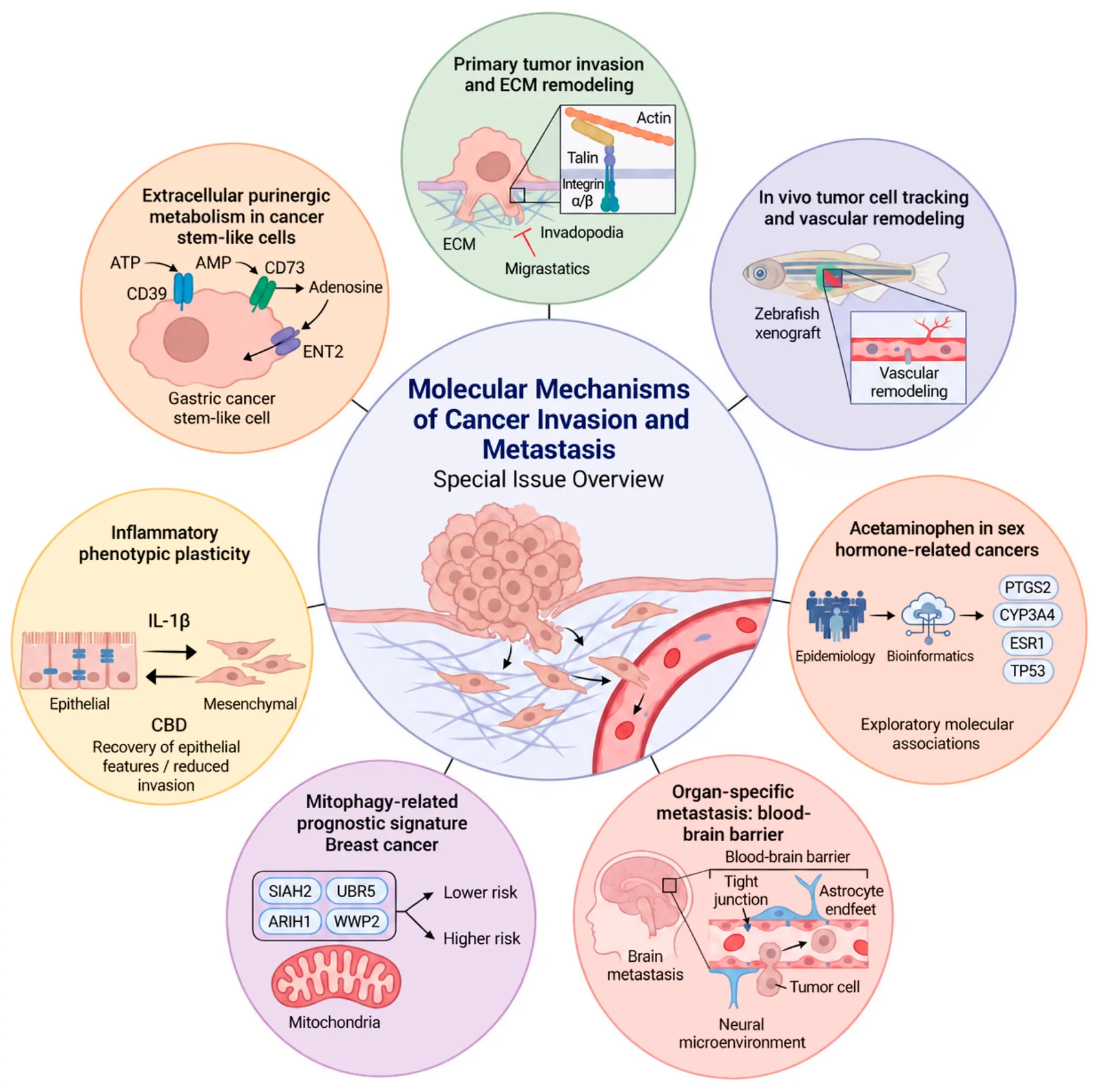
Schematic overview of the research included in this Special Issue.
